# Reference intervals for selected serum biochemistry analytes in cheetahs (*Acinonyx jubatus*)

**DOI:** 10.4102/jsava.v87i1.1316

**Published:** 2016-02-26

**Authors:** Gavin C. Hudson-Lamb, Johan P. Schoeman, Emma H. Hooijberg, Sonja K. Heinrich, Adrian S.W. Tordiffe

**Affiliations:** 1 Department of Companion Animal Clinical Studies, University of Pretoria, South Africa; 2 Institute for Zoo and Wildlife Research, Berlin, Germany; 3 Department of Research and Scientific Services, National Zoological Gardens of South Africa, South Africa

## Abstract

Published haematologic and serum biochemistry reference intervals are very scarce for captive cheetahs and even more for free-ranging cheetahs. The current study was performed to establish reference intervals for selected serum biochemistry analytes in cheetahs. Baseline serum biochemistry analytes were analysed from 66 healthy Namibian cheetahs. Samples were collected from 30 captive cheetahs at the AfriCat Foundation and 36 free-ranging cheetahs from central Namibia. The effects of captivity-status, age, sex and haemolysis score on the tested serum analytes were investigated. The biochemistry analytes that were measured were sodium, potassium, magnesium, chloride, urea and creatinine. The 90% confidence interval of the reference limits was obtained using the non-parametric bootstrap method. Reference intervals were preferentially determined by the non-parametric method and were as follows: sodium (128 mmol/L – 166 mmol/L), potassium (3.9 mmol/L – 5.2 mmol/L), magnesium (0.8 mmol/L – 1.2 mmol/L), chloride (97 mmol/L – 130 mmol/L), urea (8.2 mmol/L – 25.1 mmol/L) and creatinine (88 µmol/L – 288 µmol/L). Reference intervals from the current study were compared with International Species Information System values for cheetahs and found to be narrower. Moreover, age, sex and haemolysis score had no significant effect on the serum analytes in this study. Separate reference intervals for captive and free-ranging cheetahs were also determined. Captive cheetahs had higher urea values, most likely due to dietary factors. This study is the first to establish reference intervals for serum biochemistry analytes in cheetahs according to international guidelines. These results can be used for future health and disease assessments in both captive and free-ranging cheetahs.

## Introduction

The cheetah (*Acinonyx jubatus*) is the last remaining member of the genus *Acinonyx* and is listed as vulnerable on the International Union for Conservation of Nature (IUCN) Red List of Threatened Species (IUCN 2014). Perspectives gleaned from veterinary science can be incorporated as part of a multidisciplinary approach to conservation and can assist in the successful planning, implementation and evaluation of conservation projects (Karesh & Cook 1995). The measurement of blood analytes forms a major role in the assessment of the health of an individual or population. Species-specific haematological and serum biochemistry reference intervals are arguably among the most powerful tools in veterinary medicine to aid in the clinical decision-making process of making diagnoses and managing disease (Friedrichs *et al*. 2012).

There are only a handful of publications that provide information on haematological and serum biochemistry analytes in cheetahs (Bechert *et al.* 2002; Caro *et al.* 1987; Depauw *et al.* 2012; Holder *et al.* 2004). These studies provide values mainly for captive cheetahs. One study also provides additional values for free-ranging cheetahs in East Africa (Caro *et al.* 1987).

In addition, unpublished data are available from the International Species Information System (ISIS). These data form part of an electronic database of animals held in zoological institutions to which member institutions contribute health and genetic data (ISIS 2002). These values are presented as means, standard deviations, and minimum and maximum values.

The reference values from both published and unpublished sources do not comply with the guidelines for the generation of reference intervals in veterinary species, as stipulated by the American Society for Veterinary Clinical Pathology (ASVCP), which in turn are based on guidelines from the International Federation of Clinical Chemistry (IFCC) and the Clinical and Laboratory Standards Institute (CLSI) (Friedrichs *et al.* 2012). In view of this, the aim of this study was to establish reference intervals according to these guidelines for selected serum biochemistry analytes in healthy cheetahs. The effect of age, sex, haemolysis and captivity-status on the serum analytes and the establishment of separate reference intervals for captive and free-ranging cheetahs were investigated.

## Materials and methods

The study population consisted of 66 cheetahs (*A. jubatus*), of which 30 were captive cheetahs and 36 were free-ranging cheetahs. During June to July 2013, the captive cheetahs were immobilised and sampled at the AfriCat Foundation, Okonjima, Namibia, during their annual veterinary health examinations. The free-ranging cheetahs were trapped, immobilised and sampled on communal and commercial farmland in the Khomas and Omaheke districts, central Namibia. Animals that were used in the study were clinically healthy as far as could be determined according to history and clinical examination.

The captive cheetahs were immobilised by remote intramuscular injection and the free-ranging cheetahs were captured in box traps placed at cheetah-marking trees and immobilised by remote intramuscular injection. Immobilisation was achieved with a combination of 0.03 mg/kg medetomidine (Medetomidine, 10 mg/mL, Kyron Laboratories, Johannesburg, South Africa, 2094) and 1.2 mg/kg zolazapam/tiletimine (Zoletil^®^, Virbac Animal Health, Centurion, South Africa, 0157) for the captive animals, and 0.08 mg/kg medetomidine and 4.5 mg/kg ketamine (Ketamine 1G, Kyron Laboratories, Johannesburg, South Africa, 2094) for the free-ranging animals, injected intramuscularly. Free-ranging cheetahs were reversed with 0.25 mg/kg atipamezole and released again.

During sample collection, the sex and age of the cheetahs were recorded. The age of the captive cheetahs was transcribed from accurate records that were available at the AfriCat Foundation. The age of the free-ranging cheetahs was estimated by using the key for body size established by Caro (1994) for East African cheetahs. This was performed by evaluating shoulder height, appearance of the mane, dental wear and physical lesions such as elbow calluses and scars.

Within 15 min of immobilisation, serum samples were obtained from the captive cheetahs by collecting 10 mL of whole blood from the jugular vein with a 20 mL syringe and 18 G needle. The blood was then transferred into serum BD Vacutainer^®^ tubes (Becton, Dickinson and Company, Woodmead, Johannesburg, South Africa, 2191) and allowed to clot for 40 min on ice. After centrifuging the samples at 1700 g for 5 min, the serum was pipetted off into 1.8 mL Cryovials^®^ (Thermo Scientific, Germiston, South Africa, 1401) and frozen at -20 °C. In the free-ranging cheetahs, blood was collected directly into serum BD Vacutainer^®^ tubes. The samples were placed on ice for between 4 and 24 h until they could be centrifuged at 400 g for 15 min. The serum was then separated from the cells and frozen at -20 °C. Haemolysis scoring of the serum samples was done by means of visual assessment and rated as 0 (no haemolysis), 1+ (mild haemolysis) and > 1+ (moderate to severe haemolysis) after centrifugation was performed. Samples remained frozen until time of transportation when they were transported within 24 h in a liquid nitrogen canister to the National Zoological Gardens of South Africa (NZG), Pretoria, South Africa. The samples were stored at -80 °C at the NZG and were then transported on dry ice within 30 min to the Clinical Pathology Laboratory, Onderstepoort Veterinary Academic Hospital, Faculty of Veterinary Science, Onderstepoort Campus, University of Pretoria, Pretoria, where all the biochemistry analyses were performed.

The Cobas Integra 400 Plus Analyser^®^ (Roche, Illovo, Johannesburg, South Africa, 1609) was used to measure serum sodium, potassium, magnesium, chloride, urea and creatinine. The quantitative determination of serum sodium, potassium and chloride was achieved using ion-selective electrodes (ISE indirect method). Serum magnesium concentrations were determined using a colorimetric method with chlorophosphonazo III. Serum urea determinations were performed by a kinetic test with urease and glutamate dehydrogenase. Serum creatinine concentrations were measured using a buffered kinetic Jaffé reaction without deproteinisation. The within-laboratory co-efficient of variation for each variable was as follows: sodium, 1.1%; potassium, 1.1%; magnesium, 2.5%; chloride, 1.8%; urea, 3.1% and creatinine, 3.4%. Both daily internal and monthly external quality control was performed on this analyser and results fell within the laboratory’s preset performance goals.

The Mann–Whitney U (MWU) test was used to determine significant differences between the captive and free-ranging cheetah populations with regard to age distribution and the serum analytes. Chi-square tests were performed on sex and haemolysis scores to determine significant differences between the captive and free-ranging cheetahs. To determine whether an association existed between the age, sex or haemolysis score with serum biochemistry test results, Spearman’s rank correlation co-efficient was calculated for age, the MWU test for sex and the Kruskal–Wallis test for haemolysis score. *P*-values < 0.05 and Spearman’s correlation co-efficient of *r*_s_ > 0.6 or < -0.6 were considered significant (Weir undated).

The establishment of the reference intervals was guided by recommendations published by the ASVCP (ASVCP 2015), based in turn on guidelines from the IFCC and the CLSI (Friedrichs *et al.* 2012). Common descriptive statistics for all serum analytes included sample size, mean, median, standard deviation, minimum and maximum values. The variables were tested for Gaussian distribution according to the Anderson–Darling test with a significance level of 5%, as well as by visual inspection using histograms and Q–Q plots. The Dixon–Reed and Tukey’s tests were used to test for and identify outliers, and emphasis was on retaining rather than deleting outliers. The 90% confidence interval of the reference limits was obtained using the non-parametric bootstrap method. Reference intervals were preferentially determined by the non-parametric method, which is independent of the distribution of the data. If non-parametric methods could not be used, reference intervals were based on a robust method, preferably after Box–Cox transformation of the data to a distribution that was Gaussian.

The statistical software that was used to analyse the data was SAS^®^, Version 9.3; Statistica^®^, Version 12; and Reference Value Advisor^®^, Version 2.1 (for Microsoft Office^®^, 2010) (Geffré *et al.* 2011).

## Results

Six serum biochemistry analytes were measured in 66 healthy captive (*n* = 30) and free-ranging (*n* = 36) cheetahs, consisting of 43 males and 23 females. There were significantly more male cheetahs in the free-ranging population (75%) than in the captive one (53%) (chi-square test, *p* = 0.0499) (Table 1). The captive cheetahs were significantly older than the free-ranging cheetahs (Mann–Whitney U test, *p* < 0.01) (Table 1), with the median age of captive cheetahs (7 years) being almost twice that of the free-ranging cheetahs sampled (4 years). There was significantly more haemolysis in the free-ranging cheetah samples compared to the captive cheetah samples (chi-square test, *p* < 0.01) (Table 2).

**TABLE 1:** Summary of sex and age distributions of captive and free-ranging Namibian cheetahs.

Summary	Distributions	Captive	Free-ranging
Sex (*n*)	Male	16	27
	Female	14	9
Age (years)	Inter-quartile range	5−11	2−6
	Mean ± SD	8.5 ± 3.0	4.0 ± 2.2
	Median	7	4

Note: Age data are reported for inter-quartile range, mean ± standard deviation (SD), and median for all animals.

**TABLE 2:** Comparison of haemolysis scores of serum samples between captive and free-ranging Namibian cheetahs.

Haemolysis score	Captive	Free-ranging	Total
0†	14	0	14
1+ ‡	14	12	26
> 1+ §	2	24	26
**Total**	**30**	**36**	**66**

†, No haemolysis

‡, mild haemolysis

§, moderate-severe haemolysis.

Visual inspection of the data and the Anderson–Darling normality test showed that four of the six variables were normally distributed (potassium, magnesium, urea and creatinine). In contrast, the distribution of data was non-Gaussian for sodium and chloride and did not show Gaussian distribution even after Box–Cox transformation. Both data sets were skewed to the left. A single outlier was excluded in the calculation of the reference intervals for sodium and chloride, based on visual inspection of the data rather than reliance on the Tukey or Dixon–Reed criteria, which only listed this value as suspect on the non-transformed data. One individual was the source of the outliers for both sodium and chloride and, specifically, a very high sodium (175.6 mmol/L) and chloride (137.8 mmol/L) concentration. No outliers were excluded from other variables.

Reference intervals of the study population were calculated using the non-parametric method for serum sodium, potassium, magnesium, chloride, urea and creatinine (Table 3) and are compared with the ISIS values for all cheetahs in the ISIS database (mean, standard deviation, and minimum and maximum values) (ISIS 2002). Partitioning criteria were applied to the captive and free-ranging cheetahs and separate reference intervals were determined using the robust method after Box-Cox transformation for all the measured serum analytes. The exception was the urea of the captive population, for which a robust method was used on untransformed data (Table 4).

**TABLE 3:** Reference intervals for six serum analytes in Namibian cheetahs in the present study and ISIS values for all cheetahs.

Serum analyte	Unit	Study population							Cheetah ISIS values (2002)				
	
		*n*	Mean	SD	Median	Min	Max	RI	*n*	Mean	SD	Min	Max
Sodium	mmol/L		65	154	7	155	124	167	128–166		1066	157	4	128	175
Potassium	mmol/L		66	4.5	0.4	4.5	3.8	5.3	3.9–5.2		1068	4.4	0.5	3.1	7.0
Magnesium	mmol/L		66	1.0	0.1	1.0	0.8	1.2	0.8–1.2		26	1.0	0.1	0.8	1.5
Chloride	mmol/L		65	120	7	122	96	130	97–130		1015	122	4	108	136
Urea	mmol/L		66	13.9	4	13.3	8.1	26.2	8.2–25.1		1105	12.9	3.2	5.3	29.6
Creatinine	μmol/L		66	175	44	172	85	303	88–288		839	212	80	53	716

Note: Standard International (SI) units used.

Min, minimum; max, maximum; SD, standard deviation; RI, reference interval.

**TABLE 4:** Means, standard deviations, medians, minimum and maximum values, and reference intervals for serum analytes in captive and free-ranging Namibian cheetahs, using the Mann—Whitney U test.

Serum analyte	Unit	Captive†							Free-ranging‡					*p*-value
	
		Mean	SD	Median	Min	Max	RI	Mean	SD	Median	Min	Max	RI	
Sodium	mmol/L		154	3	154	142	153	148–160	155	157	10	124	176	137–178§	0.02
Potassium	mmol/L	4.5	0.3	4.5	3.9	5.1	3.8–5.2	4.5	4.5	0.4	3.8	5.3	3.7–5.4	0.93
Magnesium	mmol/L	1.0	0.1	1.0	0.9	1.2	0.9–1.2	1.0	1.0	0.1	0.8	1.2	0.8–1.2	0.67
Chloride	mmol/L	122	3	122	111	126	116–129	119	122	9	96	138	103–140§	0.50
Urea	mmol/L	15.9	3.7	15.7	10.5	26.2	7.4–22.9	12.3	11.4	3.5	8.1	20.0	7.2–21.4	< 0.01
Creatinine	μmol/L	184	39	183	111	273	114–276	169	163	47	85	303	89–283	0.11

Note: Standard International (SI) units used.

SD, standard deviation; min, minimum; max, maximum; RI, reference interval.

† n = 30;

‡ n = 36;

§ n = 35 (due to outlier that was excluded).

The median sodium concentration was significantly lower and the median urea concentration was significantly higher in captive cheetahs versus free-ranging cheetahs (MWU test, *p* < 0.05) (Table 4). The median sodium concentration differed by 3 mmol/L between captive (154 mmol/L ± 3 mmol/L) and free-ranging cheetahs (157 mmol/L ± 10 mmol/L), and the median urea concentration of captive cheetahs (15.7 mmol/L ± 3.7 mmol/L) was higher than that of free-ranging cheetahs (11.4 mmol/L ± 3.5 mmol/L) (Table 4).

## Ethical considerations

Research permits to conduct this study were obtained by the University of Pretoria’s Research Committee (V033/13) and Animal Ethics Committee (V033/13), National Zoological Gardens Research Ethics and Scientific Committee (NZG/P13/26) and the Namibian Ministry of Environment and Tourism (1846/2013, 1689/2012 and 1813/2013).

## Discussion

Most of the cheetahs in the study population were male (66.7%) (Table 1), which is attributable to the high proportion of males in the free-ranging population (76.9%). This result may possibly also be explained by the use of box traps to capture the free-ranging cheetahs; the box traps were placed at cheetah-marking trees, which are more often frequented by males than females. Similarly, in a study by Marker *et al.* (2003), the demographics of free-ranging Namibian cheetahs were found to be biased towards the capture of adult males, with 2.9 males captured for every adult female. Consequently, this skewed sex ratio was considered a sampling bias, rather than a true indication of population structure in the wild.

The age demographics of the study population also differed, with the captive population being significantly older than the free-ranging population (Table 1). The AfriCat Foundation does not breed any captive carnivores. The facility has few young captive animals because almost all of its cheetahs were acquired as orphaned cubs and have now been in captivity for a number of years. The highest peak of mortality in free-ranging cheetahs is between 5 and 6 years of age, with the maximum age recorded for a free-ranging Namibian cheetah being 12 years (Marker *et al.* 2003). This is consistent with the findings in this study, in which a small number (*n* = 4) of free-ranging cheetahs were over 6 years of age.

The serum samples from free-ranging cheetahs were significantly more haemolysed than those from captive cheetahs (Table 2). The blood collection technique, as well as the time interval between sample collection and processing, differed between the samples collected in the captive and free-ranging cheetahs. Operator-related factors such as operator experience or sample handling times (Grant 2003; Ong, Chan & Lim 2009) could explain the difference in haemolysis observed.

Despite significant differences in age, sex and haemolysis scores between captive and free-ranging cheetahs, none of these variables had any significant effect on the serum biochemistry analytes for the study population as well as the captive and free-ranging sub-groups. Separate reference intervals based on these variables were, therefore, not calculated.

Reference intervals of the study population were compared with the ISIS values for all cheetahs (ISIS 2002) (Table 3). The mean ISIS values for the measured serum analytes all fell within the reference intervals that were calculated in this study and were very similar to the mean values of the study population, except for creatinine (212 µmol/L), which was 1.2 times higher than the study population (175 µmol/L). The minimum ISIS values fell outside the respective reference interval, except for sodium and chloride. The maximum ISIS values were all above the reference intervals for the serum analytes in this study. Interpretation of these findings is complicated by the fact that the ISIS values are based on unpublished data taken from captive animals of unknown health status (ISIS 2002). Multiple samples may also be from the same individual, and analytical methods are not described. Furthermore, normality of these data is not reported, thus it is unclear whether these means and standard deviations can be used to establish reference intervals.

Separate reference intervals for the captive and free-ranging sub-groups were calculated according to ASVCP guidelines (ASVCP 2015) (Table 4). The uncertainty and imprecision of the reference limits may be very high because there were fewer than 40 samples for each subgroup (Geffré *et al.* 2009). Nevertheless, there were large differences in the reference intervals for serum sodium, chloride and creatinine, whenever these analytes had reference intervals that were wider in the free-ranging cheetahs.

The free-ranging cheetahs may have a slightly higher median sodium value than captive cheetahs because of dehydration. The free-ranging cheetahs were caught in box traps with no free access to water and variable lengths of time may have elapsed before samples were collected from them.

Pre-renal causes for elevated urea are increased protein catabolism (e.g. small bowel haemorrhage and necrosis, starvation, and prolonged exercise), dehydration and high-protein diets (Backlund *et al.* 2011; DiBartola 2010). The captive cheetahs at the AfriCat Foundation are fed a high-protein diet of 1.0 kg – 1.5 kg donkey meat daily, compared with their free-ranging counterparts that consume the muscle meat and viscera of small antelope, rodents and birds and seldom eat daily (Bechert *et al.* 2002). The high frequency of feeding large portions of high-protein meat may create an overall protein excess and elevated urea levels in comparison with free-ranging cheetahs. In a study by Caro *et al.* (1987), it was also found that captive cheetahs had higher urea levels than free-ranging cheetahs. This finding was attributed to a higher total food intake, because it was also found that free-ranging cheetahs were consuming about 75% of the captive cheetah diet. Moreover, the cheetahs in captivity have free access to water at all times, ruling out dehydration as a possible pre-renal cause of elevated urea. Other causes of increased protein catabolism are unlikely in the captive cheetah population as they were all fed daily and do not exercise for prolonged periods. The high-protein diet could, however, mask elevated urea levels due to renal disease (renal azotaemia) and/or gastritis (small bowel haemorrhage) (DiBartola 2010). Lymphoplasmacytic gastritis and renal disease are two of the most prevalent diseases of captive cheetahs and are rare in free-ranging cheetahs (Munson *et al.* 2005). Renal disease is a leading cause of morbidity and mortality in captive cheetahs and renal lesions were present in 90% of a captive cheetah population in one study (Munson 1993). Glomerulosclerosis is one of the two main lesions seen in captive cheetahs with renal disease, the other being renal amyloidosis (Bolton & Munson 1999). Over 80% of captive cheetahs in the United States and South Africa suffer from glomerulosclerosis (Munson 1993). Bolton and Munson (1999) stated that the daily feeding of a high-protein diet to captive cheetahs may influence the development of glomerulosclerosis. Clinical pathology indices of renal dysfunction, such as serum creatinine and urea levels, are therefore highly relevant and essential in the detection of renal disease in cheetahs, particularly captive ones.

### Limitations of the study

In this study, several limitations need to be considered. The size of the study population was a major limitation. This data set contains only captive cheetahs from the AfriCat Foundation and free-ranging cheetahs from the Khomas and Omaheke districts in Namibia and the reference intervals generated apply to these populations and the analytical methods used. Cheetahs from other locations may have somewhat different serum reference values. Reference intervals may also differ based on the analytical methods used. Furthermore, due to the high prevalence of renal disease in captive cheetahs, particularly glomerulosclerosis, there may be individuals in the study population suffering from renal disease which will influence the reference intervals calculated. In order to use these reference intervals for other populations, transference or validation procedures should be performed according to CLSI and IFCC standards (Geffré *et al.* 2009).

Future studies may benefit from a larger study population, and from wider and/or multiple geographical location(s). Furthermore, reference intervals for only a selection of serum biochemistry analytes were established. The establishment of reference intervals for haematological and other various serum biochemistry analytes is recommended.

## Conclusion

This is the first report of baseline reference intervals for serum biochemistry analytes of healthy captive and free-ranging cheetahs in Namibia based on the guidelines stipulated by the IFCC and CLSI regarding the generation of reference intervals for veterinary species. These reference intervals can be considered a useful tool for veterinary practitioners, researchers, zoologists and any other professionals working in cheetah medicine and conservation. These results will aid in the accurate assessment of health and the management of disease in cheetahs. Further research into the pathophysiology of diseases affecting cheetahs, using comprehensive health assessment data, is required to fully understand the threats facing this vulnerable species.
